# Characterization of miRNAs from sardine (*Sardina pilchardus* Walbaum, 1792) and their tissue-specific expression analysis in brain and liver

**DOI:** 10.1007/s13205-020-02298-y

**Published:** 2020-06-26

**Authors:** Juan Luis de la Fuente Jiménez, Ashutosh Sharma, Sujay Paul

**Affiliations:** School of Engineering and Sciences, Tecnologico de Monterrey, Campus Queretaro, Av. Epigmenio Gonzalez, No. 500 Fracc. San Pablo, 76130 Queretaro, Mexico

**Keywords:** Sardine (sardina pilchardus walbaum, 1792), microRNAs, Computational identification, qRT-PCR analysis, Minimum folding free energy index (MFEI)

## Abstract

MicroRNAs are endogenous highly conserved short (~ 21 nucleotides) non-coding RNA molecules that play key roles in post-transcriptional gene regulation by translational inhibition or by target mRNA cleavage. In this report, using high stringent computational-based methods, a total of 101 putative miRNAs were identified from European sardine fish (*Sardina pilchardus* Walbaum, 1792). All the precursors of identified sardine miRNAs formed stable stem-loop structures and displayed high minimum free energy index (MFEI) values. For the experimental validation of the computationally predicted miRNAs, a tissue-specific quantitative study of eight randomly selected putative sardine miRNAs (*spi-miR9, spi-miR26, spi-miR128, spi-miR129, spi-miR132, spi-miR212, spi-miR219, and spi-miR338*) was performed in brain and liver and all the selected miRNAs were found to be overexpressed in brain tissue. Moreover, using RNAhybrid, a total of 83 potential target proteins of the characterized sardine miRNAs were identified those are involved in transcription, cellular development, defense mechanism, and various signaling pathways. To the best of our knowledge, this is the first report of sardine microRNAs and their targets.

**Electronic supplementary material:**

The online version of this article (10.1007/s13205-020-02298-y) contains supplementary material, which is available to authorized users.

## Introduction

European sardine (*Sardina pilchardus* Walbaum, 1792), commonly known as European pilchard or simply sardine is one of the most abundant small pelagic fish in the world from the Clupeidae family that occurs mostly in the Atlantic Ocean and the Mediterranean Sea (Louro et al. 2019) and widely consumed by humans. Due to the richest and cheapest source of healthy omega-3 fatty acids, over the last fifty years, the global capture of the sardine has raised from 610,438 t in 1970 up to 1,281,391 t in 2016 representing a significant fish species for fisheries (https://www.fao.org/fishery/species/2910/en). Besides the commercial importance, sardine also plays a key role in the food chain of the marine ecosystem by connecting the first producers of energy to the top of the trophic chain reaching predators. Although European sardine is considered in a global scope assessment with a conservation status of least concern species in the International Union for Conservation of Nature and Natural Resources red list of threatened species (https://www.iucnredlist.org/species/198580/15542481), recently, marine biologists have warned that due to overfishing, pollution, habitat damage, climate change, and various diseases sardine population is declining rapidly worldwide facing the threat of extinction in near future (https://www.express.co.uk/news/nature/756417/Sardines-extinction-threat-overfishing-wiped-outAtlantic-fish-onservationists). Nevertheless, current advancement in molecular technologies greatly facilitates the use of genomics or transcriptomics knowledge to develop modern and rapid monitoring tools for endangered or threatened biodiversity.

MicroRNAs (miRNAs) are small endogenous ~ 21-nucleotide (nt) long non-coding regulatory RNA molecules that play a pivotal role in gene expression at the post-transcriptional level. It has been evidenced that miRNAs regulate a wide variety of biological processes such as cell cycle control, cell proliferation and differentiation, organ development, apoptosis, and stress response signaling in both animals and plants (Sun and Lai 2013; Paul et al. 2011, 2020). Moreover, highly tissue-specific expression patterns during embryogenesis suggest that microRNAs also play an important role in the differentiation and maintenance of tissue identity (Ribeiro et al. 2014). The evolutionarily conserved sequences of miRNAs across different species simplify the characterization process of new miRNA orthologues through computational based homology analysis (Sharma et al. 2019); however, in silico miRNA identification only based on sequence similarity generates false-positive results and hence other stringent parameters of the predicted miRNA precursors such as minimum folding free energy (MFE), sequence length, GC content, and the minimum folding free energy index (MFEI) are required to increase the prediction precision (Paul et al. 2018). Though, experimental validation of the predicted miRNAs is a crucial step to authenticate the prediction (Sharma et al. 2019).

Nonetheless, due to the rapid increment of the human population marine ecosystem are progressively exposing to numerous anthropogenic stressors resulting in a negative impact on biodiversity. This emphasizes the need to assess the effects of stressors on aquatic organisms so that the regulation could be tighter before irreversible damages occur to the marine communities. Thus, precise molecular approaches such as using microRNAs as a biomarker to study the effect of anthropogenic stressors to the aquatic communities could be an indicator for the policymaker to make their decision regarding the conservation of a species (Ikert and Craig 2020). Moreover, few miRNAs such as miR-9, miR-128, miR-129, miR132, and miR-219 are expressed in the brain providing a more effective knowledge of the environment, physiology, and for understanding the molecular mechanism involved in teleost fishes giving baseline information for commercial and conservation tasks (Subramanian et al. 2017; Xu et al. 2017; Bizuayehu and Babiak 2014). Nevertheless, since miRNAs play various regulatory roles and take part in a wide variety of biological processes it is important to exploit the recently published sardine genome information (GenBank accession UIGZ00000000; Louro et al. 2019) to gain a better understanding about the physiological role of miRNAs in sardine. Furthermore, recently, chemically modified antisense oligonucleotides (antimiRs), which sequester the mature miRNAs in competition with cellular target mRNAs leading to the functional inhibition of the miRNAs and de-repression of the direct targets, have been successfully employed in vivo, including in zebrafish (Stenvang et al. 2012). We believe that in near future current sardine miRNA information will help for the development of stress biomarkers as well as facilitate antimiR research to counteract biotic and abiotic stress-related disorders in sardine and other fishes. In summary, to increase knowledge about miRNAs and their functions in a commercially valuable popular fish sardine we aimed to characterize the unknown microRNAs and their targets in sardine and explore their tissue-specific expression pattern through a quantitative approach.

## Material and methods

### Computational prediction of sardine miRNA

For the in silico prediction of potential sardine miRNAs, two different reference sets of mature fish miRNA sequences were obtained from the miRbase miRNA database (https://www.mirbase.org/cgi-bin/browse.pl) and aligned with the whole genome sequence (WGS) of sardine. The reference set comprised a total of 889 mature miRNAs sequences including 373 mature sequences from vertebrate model fish *Danio rerio* (dre) or Zebrafish and 516 mature sequences from popular fish cod *Gadus morhua* (gmo). The alignment between the reference set of mature miRNAs and the WGS of sardine was done with the BLASTn tool and the sequences that showed the exact match were chosen manually. The potential precursor (pre-miRNA) sequences of nearly 400 nt (200 nt downstream and 200 nt upstream of the hit region from BLAST) were mined and sequences coding for proteins were eliminated. To check the reliability of the potential precursors, the secondary structures were predicted using the MFOLD web server (https://unafold.rna.albany.edu/?q=mfold). Since the stable secondary structure of the precursors is considered as one of the important factors to be a miRNA candidate some previously demonstrated strict filtering criteria were applied during secondary structure prediction such as: (1) the precursors must form a stem‐loop structure containing mature miRNA sequences within one arm (2) the potential miRNA sequences should not be positioned at the terminal loop of the hairpin structures, (3) mature miRNAs should have fewer than nine mismatches with the opposite miRNA^*^ sequence, and (4) the predicted secondary structures must have low MFE and high MFEI values since it is required for distinguishing the miRNAs from other RNAs molecules (MFEIs of tRNAs, rRNAs or mRNAs candidates are 0.64, 0.59 and 0.62–0.66, respectively) (Zhang et al. 2006). The MFE or ΔG (-kcal/mol) values generated from the MFOLD web server of the stem-loop structures were used to calculate the MFEI values using the following formula:

$${\rm{MFEI}} = {\rm{ }}{{{\rm{(MFE}}/{\rm{length \, of \,  RNA \,sequence)}} \times 100\% } \over {{\rm{GC \, content}}}}$$

### Prediction of sardine miRNA targets and their functional annotation

The near precise complementarity between miRNAs and their target sequences enabled in silico prediction of potential target transcripts in sardine. In this report, the potential target transcripts of sardine miRNAs were initially predicted using the NCBI BLASTn program by subjecting the mature miRNA sequences as queries. The Reference RNA sequence database (rfseq_rna) of teleost fishes was chosen during the BLAST analysis. The mRNA sequences with ≥ 75% of query coverage as well as the percent identity were selected for further analysis by RNA-hybrid program (Krüger and Rehmsmeier 2006), and the parameters used are defined as follows: (1) no mismatches at 2–8 nt position (seed region) of mature miRNA with its complementary sequence, (2) only one G:U pairing in the seed region, and (3) no more than four gaps in miRNA from the 9 nt to 21 nt. To achieve a better comprehension functional annotation of the predicted targets was performed using the AmiGO2 platform (https://amigo.geneontology.org/amigo/dd_browse).

### RNA extraction and tissue-specific miRNA expression analysis

Five frozen adult sardine fish samples size ranging from 19 to 23 cm were used for total RNA including small RNA extraction from liver and brain tissues using miRNeasy Mini Kit (Qiagen) and pooled separately for each tissue type. The quality and quantity of RNA samples were measured with Nanodrop One (Thermo Scientific, Wilmington, USA), and subsequently polyadenylated (using modified oligo dT primer) as well as reverse transcribed using mRQ Buffer (2X) and enzyme provided with Mir-X miRNA First-Stand Synthesis kit (Takara, Tokyo, Japan). In this study, 1 µg of total RNA (including small RNAs) was used for reverse transcription reaction. Randomly selected eight sardine microRNAs (*spi-miR-9-3p, spi-miR-26a-5p, spi-miR-128-3p, spi-miR-129-1-3p, spi-miR132-3p, spi-miR-212, miR219-3p, spi-miR-338*) were experimentally validated and their tissue-specific expression pattern in brain and liver was checked using Step One Real-Time PCR System (Applied Biosystems, Carlsbad, CA) and Mir-X miRNA TB Green qRT-PCR kit (Takara, Tokyo, Japan). The real-time qRT-PCR reaction was made in a volume of 12.5 µl containing 1X TB Green Advantage Premix, 1X ROX Dye, 0.2 µm each of forward and reverse primers, and 0.5 µl of cDNA. U6 was employed as an internal reference and each reaction was done in three technical replicates. The qRT-PCR program was as follows: initial denaturation for 10 s at 95 °C, then 45 cycles of denaturation for 5 s at 95 °C and annealing for 20 s at 60 °C. This cycle was followed by a melting curve analysis ranging from 55 to 95 °C, with temperature increasing steps of 0.5 °C every 10 s. Melting curves for each amplicon were observed carefully to confirm the specificity of the primers used. Finally, the relative fold change values were obtained using the comparative C_t_ method or Ct (2^−ΔΔCT^).

## Results and discussion

### Characterization of sardine miRNAs and their tissue-specific expression analysis

In this report using strict filtering criteria, a total of 101 potentially conserved sardine miRNAs were identified (Table 1). The majority of the identified sardine miRNAs were 22 nucleotides (nt) long while their precursors displayed great size variability ranging between 53 and 116 nt with an average of 62 nt (Table 1). Regarding the miRNA location, 64.4% of the putative sardine miRNAs were found located at the 3′ arm of the stem-loop precursors, while the remaining 35.6% were located at the 5′ arm. Moreover, 59% of the predicted sequences began with the uracil (U) nucleotide corroborating the study of Zhang et al. (2008) that miRNA mediated regulation is highly dependent on U existing at the initial position of the mature miRNA sequence. The content of guanine-cytosine (GC) of sardine miRNA precursors had an average of 44.90%. It is well known that low MFE values of the stem-loop precursors attain more stable miRNA predictions (Bonnet et al. 2004), in this study, quite low MFE values of the precursors varied from − 16.10 to − 46.80 with an average of − 25.50, was successfully achieved. Moreover, the MFEI scores ranged from 0.70 to 1.33 with a mean of 0.93 excluding the chance of being another small RNA. The predicted stem-loop secondary structures of sardine’s miRNA precursors pre-miRNA with higher MFEI values (top 20) were displayed in supplementary file 1.

**Table 1:** Summary of the identified *Sardina pilchardus* (Walbaum, 1792) miRNAs

Identified miRNAs	LM (nt)	Query miRNAs	miRNA sequences	Location	LP (nt)	GC %	MFEs	MFEI
*spi-miR-1*	22	dre-miR-1	UGGAAUGUAAAGAAGUAUGUAU	3′	64	29.69	− 24.00	1.26
*spi-let-7a*	22	dre-let-7a	UGAGGUAGUAGGUUGUAUAGUU	5′	70	37.14	− 28.80	1.10
*spi-miR-9-3p*	21	dre-miR-9-3p	UAAAGCUAGAUAACCGAAAGU	3′	60	38.33	− 26.70	1.16
*spi-miR-10a-5p*	22	dre-miR-10a-5p	UACCCUGUAGAUCCGAAUUUGU	5′	61	45.90	− 21.60	0.77
*spi-miR-15a-3p*	22	dre-miR-15a-3p	CAGGCCGUACUGUGCUGCGGCA	3′	61	57.38	− 29.60	0.84
*spi-miR-16a*	22	dre-miR-16a	UAGCAGCACGUAAAUAUUGGUG	5′	65	38.46	− 22.70	0.90
*spi-miR-17a-2-3p*	23	dre-miR-17a-2-3p	ACUGCAGUGGAGGCACUUCAAGC	3′	63	47.62	− 21.50	0.71
*spi-miR-19a-3p*	23	dre-miR-19a-3p	UGUGCAAAUCUAUGCAAAACUGA	3′	58	36.21	− 23.60	1.12
*spi-miR-20a-3p*	22	dre-miR-20a-3p	ACUGCAGUGUGAGCACUUGAAG	3′	60	38.33	− 24.70	1.07
*spi-miR-22a-3p*	22	dre-miR-22a-3p	AAGCUGCCAGCUGAAGAACUGU	3′	65	46.15	− 32.50	1.08
*spi-miR-23a-3p*	22	dre-miR-23a-3p	AUCACAUUGCCAGGGAUUUCCA	3	60	45.26	− 30.10	1.10
*spi-miR-24*	22	dre-miR-24	UGGCUCAGUUCAGCAGGAACAG	3′	58	56.9	− 23.90	0.72
*spi-miR-25-3p*	22	dre-miR-25-3p	CAUUGCACUUGUCUCGGUCUGA	3′	62	54.84	− 24.10	0.70
*spi-miR-26a-5p*	22	dre-miR-26a-5p	UUCAAGUAAUCCAGGAUAGGCU	5′	59	49.15	− 27.00	0.93
*spi-miR-27a-3p*	22	dre-miR-27a-3p	UUCACAGUGGCUAAGUUCCGCU	3	67	48.19	− 24.0	0.74
*spi-miR-29a*	22	dre-miR-29a	UAGCACCAUUUGAAAUCGGUUA	3′	62	45.73	− 27.50	0.96
*spi-miR-30b*	22	dre-miR-30b	UGUAAACAUCCUACACUCAGCU	5′	57	49.12	− 24.40	0.87
*spi-miR-31*	22	dre-miR-31	UGGCAAGAUGUUGGCAUAGCUG	5′	58	51.72	− 27.00	0.90
*spi-miR-33b-5p*	21	gmo-miR-33b-5p	GUGCAUUGUAGUUGCAUUGCA	5′	57	43.86	− 28.20	1.12
*spi-miR-34a*	22	dre-miR-34a	UGGCAGUGUCUUAGCUGGUUGU	5′	63	49.21	− 28.30	0.91
*spi-miR-92a-3p*	22	dre-miR-92a-3p	UAUUGCACUUGUCCCGGCCUGU	3′	62	53.23	− 35.50	1.07
*spi-miR93*	22	dre-miR93	AAAAGUGCUGUUUGUGCAGGUA	5′	59	47.46	− 25.40	0.90
*spi-miR-96-3p*	22	dre-miR-96-3p	CAAUUAUGUGUAGUGCCAAUAU	3′	66	34.85	− 25.70	1.11
*spi-miR-100–2-3p*	22	dre-miR-100–2-3p	CAAGCUCGUGUCUAUAGGUAUG	3′	59	49.29	− 22.40	0.77
*spi-miR-101a*	22	dre-miR-101a	UACAGUACUGUGAUAACUGAAG	3′	61	40.98	− 28.50	1.14
*spi-miR-103*	23	dre-miR-103	AGCAGCAUUGUACAGGGCUAUGA	3′	61	50.82	− 25.30	0.81
*spi-miR-122*	22	dre-miR-122	CAAACACCAUUGUCACACUCCA	3′	58	41.67	− 25.30	1.04
*spi-miR-124-3p*	22	dre-miR-124-3p	UAAGGCACGCGGUGAAUGCCAA	3′	61	45.90	− 23.10	0.82
*spi-miR-125a*	22	dre-miR-125a	UCCCUGAGACCCUUAACCUGUG	5′	56	51.79	− 25.80	0.88
*spi-miR-126a-3p*	21	dre-miR-126a-3p	UCGUACCGUGAGUAAUAAUGC	3′	59	42.37	− 19.70	0.78
*spi-miR-128-3p*	22	dre-miR-128-3p	UCACAGUGAACCGGUCUCUUUU	3′	53	50.94	− 19.50	0.72
*spi-miR-129–1-3p*	21	dre-miR-129–1-3p	GAAGCCCUUACCCCAAAAAGU	3′	65	51.54	− 24.70	0.73
*spi-miR-130c-3p*	22	dre-miR-130c-3p	CAGUGCAAUAUUAAAAGGGCAU	3′	61	39.34	− 25.60	1.06
*spi-miR132-3p*	22	dre-miR132-3p	UAACAGUCUACAGCCAUGGUCG	3′	63	52.38	− 30.50	0.92
*spi-miR133a-2-5p*	21	dre-miR133a-2-5p	AGCUGGUAAAAUGGAACCAAA	5′	58	44.83	− 22.00	0.84
*spi-miR-135a*	23	dre-miR-135a	UAUGGCUUUUUAUUCCUAUGUGA	5′	60	40.00	− 24.10	1.00
*spi-miR-138–2-3p*	21	dre-miR-138–2-3p	GCUUCUUCACAACACCAGGGU	3′	62	56.45	− 29.90	0.85
*spi-miR-140-3p*	23	dre-miR-140-3p	UACCACAGGGUAGAACCACGGAC	3′	66	53.03	− 33.50	0.95
*spi-miR-142a-3p*	23	dre-miR-142a-3p	UGUAGUGUUUCCUACUUUAUGG	3′	60	41.67	− 25.00	0.99
*spi-miR-143*	21	dre-miR-143	UGAGAUGAAGCACUGUAGCUC	3′	57	50.88	− 26.60	0.91
*spi-miR-144-3p*	20	dre-miR-144-3p	UACAGUAUAGAUGAUGUACU	3′	59	30.51	− 21.30	1.18
*spi-miR-145-3p*	22	dre-miR-145-3p	GGAUUCCUGGAAAUACUGUUCU	3′	63	47.62	− 27.80	0.92
*spi-miR-146b*	22	dre-miR-146b	UGAGAACUGAAUUCCAAGGGUG	5′	58	48.28	− 22.60	0.80
*spi-miR-148*	22	dre-miR-148	UCAGUGCAUUACAGAACUUUGU	3′	62	40.32	− 24.20	0.96
*spi-miR-150*	22	dre-miR-150	UCUCCCAAUCCUUGUACCAGUG	5′	59	54.24	− 31.80	0.99
*spi-miR-152-3p*	21	gmo-miR-152-3p	CAAAGUUCUGUUAUGCACUGA	5′	61	39.34	− 21.90	0.91
*spi-miR-153a-3p*	22	dre-miR-153a-3p	UUGCAUAGUCACAAAAGUGAUC	3′	62	37.1	− 24.40	1.06
*spi-miR-155*	22	dre-miR-155	UUAAUGCUAAUCGUGAUAGGGG	5′	59	35.59	− 22.10	1.05
*spi-miR-183-5p*	23	dre-miR-183-5p	UAUGGCACUGGUAGAAUUCACUG	5′	60	40.00	− 20.70	0.86
*spi-miR-184*	22	dre-miR-184	UGGACGGAGAACUGAUAAGGGC	3′	64	48.44	− 22.60	0.72
*spi-miR-185a-5p*	23	dre-miR-185a-5p	AACAUUCAACGCUGUCGGUGAGU	5′	61	44.26	− 20.40	0.75
*spi-miR-187*	20	dre-miR-187	UCGUGUCUUGUGUUGCAGCC	3′	60	63.33	− 39.50	1.03
*spi-miR-190a*	22	dre-miR-190a	ACCUAAUAUAUCAAACAUAUCA	3′	57	26.32	− 18.00	1.19
*spi-miR-192*	21	dre-miR-192	AUGACCUAUGAAUUGACAGCC	5′	61	47.54	− 23.90	0.82
*spi-miR-193a-3p*	22	dre-miR-193a-3p	AACUGGCCUACAAAGUCCCAGU	3′	62	46.77	− 23.40	0.80
*spi-miR-194a*	21	dre-miR-194a	UGUAACAGCAACUCCAUGUGG	5′	55	47.27	− 27.90	1.07
*spi-miR-196a-5p*	22	dre-miR-196a-5p	UAGGUAGUUUCAUGUUGUUGGG	5′	60	36.67	− 21.00	0.95
*spi-miR-199-3p*	22	dre-miR-199-3p	UACAGUAGUCUGCACAUUGGUU	3′	61	49.18	− 23.90	0.79
*spi-miR-200a-3p*	22	dre-miR-200a-3p	UAACACUGUCUGGUAACGAUGU	3′	61	45.9	− 23.90	0.85
*spi-miR-202-5p*	22	dre-miR-202-5p	UUCCUAUGCAUAUACCUCUUUG	5′	56	42.86	− 22.60	0.94
*spi-miR-203a-3p*	22	dre-miR-203a-3p	CAAGUGGUCCUAAACAUUUCAC	5′	62	40.32	− 18.20	0.72
*spi-miR-204-5p*	22	dre-miR-204-5p	UUCCCUUUGUCAUCCUAUGCCU	5′	56	53.57	− 26.50	0.88
*spi-miR-205-5p*	22	dre-miR-205-5p	UCCUUCAUUCCACCGGAGUCUG	5′	57	45.61	− 25.20	0.96
*spi-miR-206-3p*	22	dre-miR-206-3p	UGGAAUGUAAGGAAGUGUGUGG	3′	62	41.94	− 30.20	1.16
*spi-miR-212*	21	dre-miR-212	UAACAGUCUACAGUCAUGGCU	3′	57	50.75	− 24.50	0.84
*spi-miR-214*	21	dre-miR-214	CUGCCUGUCUGUGCCUGCUGU	5′	62	56.45	− 36.80	1.05
*spi-miR-216a*	22	dre-miR-216a	UAAUCUCAGCUGGCAACUGUGA	5′	60	53.33	− 26.00	0.81
*spi-miR-217*	22	dre-miR-217	UACUGCAUCAGGAACUGAUUGG	5′	59	42.37	− 33.40	1.33
*spi-miR-218a*	22	dre-miR-218a	UUGUGCUUGAUCUAACCAUGUG	5′	63	55.56	− 27.10	0.77
*spi-miR219-3p*	23	dre-miR219-3p	GGAGUUGUGGAUGGACAUCACGC	3′	68	47.06	− 30.20	0.94
*spi-miR-221-3p*	23	dre-miR-221-3p	AGCUACAUUGUCUGCUGGGUUUC	3′	65	43.08	− 29.60	1.05
*spi-miR-222a-3p*	24	dre-miR-222a-3p	AGCUACAUCUGGCUACUGGGUCUC	3′	64	50.00	− 28.40	0.88
*spi-miR-223*	21	dre-miR-223	UGUCAGUUUGUCAAAUACCCC	3′	63	44.44	− 24.60	0.87
*spi-miR-338*	22	dre-miR-338	UCCAGCAUCAGUGAUUUUGUUG	3′	61	44.26	− 22.50	0.83
*spi-miR-363-3p*	22	dre-miR-363-3p	AAUUGCACGGUAUCCAUCUGUA	3′	76	34.21	− 23.50	0.90
*spi-miR-365*	22	dre-miR-365	UAAUGCCCCUAAAAAUCCUUAU	3′	65	46.15	− 28.40	0.94
*spi-miR-429a*	22	dre-miR-429a	UAAUACUGUCUGGUAAUGCCGU	3′	64	40.63	− 26.90	1.03
*spi-miR-430a-3p*	22	dre-miR-430a-3p	UAAGUGCUAUUUGUUGGGGUAG	3′	61	44.26	− 21.00	0.77
*spi-miR-454b*	22	dre-miR-454b	UAGUGCAAUAUUGCUUAUAGGG	3′	63	38.1	− 23.10	0.96
*spi-miR-455-3p*	23	dre-miR-455-3p	GUGUAUAUGCCCAUGGACUGCAU	5′	62	53.23	− 34.30	1.03
*spi-miR-456*	22	dre-miR-456	CAGGCUGGUUAGAUGGUUGUCA	3′	65	52.31	− 28.80	0.84
*spi-miR-458-3p*	22	dre-miR-458-3p	AUAGCUCUUUGAAUGGUACUGC	3′	60	48.33	− 31.60	1.08
*spi-miR-459-5p*	22	dre-miR-459-5p	UCAGUAACAAGGAUUCAUCCUG	5′	61	45.90	− 25.50	0.91
*spi-miR-460-3p*	22	dre-miR-460-3p	CACAGCGCAUACAAUGUGGAUG	3′	61	44.26	− 20.00	0.74
*spi-miR-462*	22	dre-miR-462	UAACGGAACCCAUAAUGCAGCU	5′	116	50.00	− 46.80	0.80
*spi-miR-489*	23	dre-miR-489	AGUGACAUCAUAUGUACGGCUGC	3′	61	42.62	− 27.20	1.04
*spi-miR-499-3p*	22	dre-miR-499-3p	AACAUCACUUUAAGUCUGUGCU	3′	62	38.71	− 22.80	0.94
*spi-miR-722*	24	dre-miR-722	UUUUUUGCAGAAACGUUUCAGAUU	3′	67	31.34	− 21.40	1.01
*spi-miR-724*	22	dre-miR-724	AACAGUCGCAAAUUCCCUUUAA	3′	58	37.93	− 21.70	0.98
*spi-miR-726*	21	dre-miR-726	UUCACUACUAGCAGAACUCGG	3′	63	42.86	− 19.20	0.71
*spi-miR-727-3p*	22	dre-miR-727-3p	GUUGAGGCGAGUUGAAGACUUA	3′	65	47.69	− 22.50	0.72
*spi-miR-728*	22	dre-miR-728	AUACUAAGUACACUACGUUUUC	3′	66	33.33	− 21.30	0.96
*spi-miR-729*	24	dre-miR-729	CAUGGGUAUGAUACGACCUGGGUU	3′	64	43.75	− 29.20	1.04
*spi-miR-734-3p*	21	gmo-miR-734-3p	UAAAUGCUGCAGAAUUGUGCU	3′	60	38.33	− 16.10	0.70
*spi-miR-737-3p*	21	dre-miR-737-3p	AAUCAAAACCUAAAGAAAAUA	3′	64	28.13	− 20.70	1.14
*spi-miR-1788-3p*	21	dre-miR-1788-3p	CAGGCAGCUAAAGCAAGUCUG	3′	60	48.33	− 29.20	1.00
*spi-miR-2187-3p*	22	dre-miR-2187-3p	UUACAGGCUAUGCUAAUCUAUG	3′	63	30.16	− 23.80	1.25
*spi-miR-2188-5p*	21	dre-miR-2188-5p	AAGGUCCAACCUCACAUGUCC	5′	56	57.14	− 26.30	0.82
*spi-miR-7552-5p*	22	gmo-miR-7552-5p	UUACAAUUAAAGGAUAUUUCUU	5′	60	30.00	− 17.90	0.99
*spi-miR-8160b-5p*	21	gmo-miR-8160b-5p	AGAAUAAUGCCAGCAGUCGGC	5′	56	51.79	− 22.50	0.77
*spi-miR-10545-5p*	22	gmo-miR-10545-5p	UAAGUCUCACACCAGUGCAAAA	5′	56	46.30	− 19.60	0.75

*LM* length of miRNA, *LP* length of precursor, *GC* guanine-cytosine, *MFE* minimum folding free energy, *MFEI* minimum folding free energy index

**Fig. 1:**
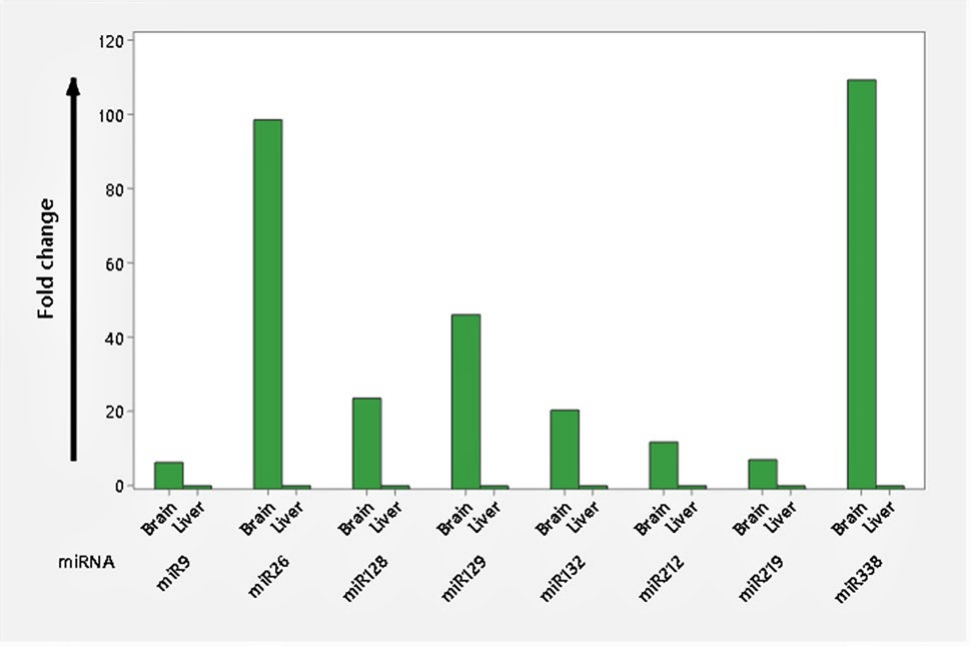
Graphical representation of the fold changes (differential expression pattern) of selected *Sardina pilchardus* (Walbaum, 1792) microRNAs (*spi-miR9, spi-miR26, spi-miR128, spi-miR129, spi-miR132, spi-miR212, spi-miR219, and spi-miR338*) between brain and liver tissue samples. U6 was chosen as an internal reference. All the miRNAs were found to be brain-specific and overexpressed in brain tissue. MicroRNA *spi-miR338* was the highest expressed miRNAs among all followed by *spi-miR26*

Randomly selected eight sardine miRNAs (*spi-miR9, spi-miR26, spi-miR128, spi-miR129, spi-miR132, spi-miR212, spi-miR219, and spi-miR338*) were successfully validated in this study by qRT-PCR and their significant differential expression between brain and liver tissues was noticed. Interestingly, all the selected sardine miRNAs were overexpressed in the brain as compared to the liver. The expression of *spi-miR338*, *spi-miR26*, and *spi-miR129* had the high fold changes of 109.13, 98.36, and 45.93, respectively, while *spi-miR128* and *spi-miR132* showed near similar fold changes of 23.50 and 20.35, respectively. However, *spi-miR212*, *spi-miR129*, and *spi-miR9* exhibited the lowest fold changes with the values of 11.10, 7.00, and 6.23, respectively (Fig. 1). Since individual functions of those selected miRNAs are not well studied in teleost fish, we explored their function in other vertebrates, and it was revealed that all of them are brain-enriched miRNAs and participate in several neurological functions in other vertebrate species, including human. For example, miR9 was found to be one of the most highly expressed microRNAs in the developing and adult vertebrate brain and participate mainly in neural differentiation proliferation, differentiation, and cell migration (Coolen et al. 2013); while miR26 regulates neural stem cell development and targets brain-derived neurotrophic factor proteins involved in plasticity and synaptogenesis (Caputo et al. 2011). Similarly, miR129, miR212, and miR132 are also found to be involved in synaptic plasticity (Follert et al. 2014; Thangaleela et al. 2018); while miR219 was reported to endorse neural precursor cell differentiation (Murai et al. 2016). Likewise, brain enriched miR128 and miR338 are involved in neuronal cell migration and oligodendrocytes development, respectively (Evangelisti et al. 2009; Follert et al. 2014).

### Identification of potential target transcripts of putative sardine miRNAs

**Fig. 2:**
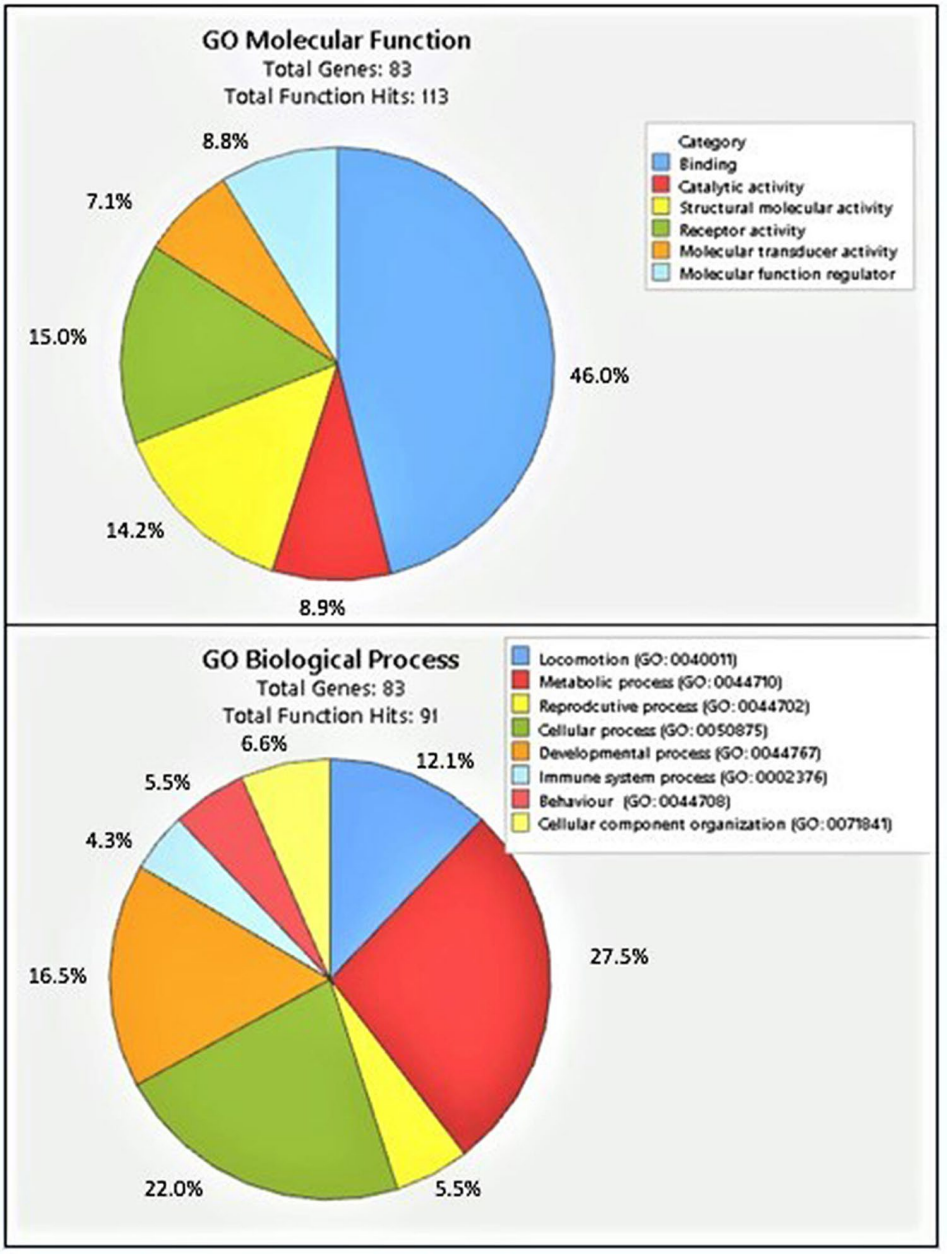
Functional annotation of potential *Sardina pilchardus* (Walbaum, 1792) target transcripts. Most of the predicted targets were found to be involved in binding (46%) in the molecular function category, while in the biological process category most of the targets are involved in metabolic processes (27.5%)

In this report, a total of 83 potential target transcripts of sardine miRNAs were identified, and among them, several microRNAs were found to target more than one transcript (Supplementary File 1). Most of the sardine miRNA targets identified in this study are involved in transcription, cellular development, defense mechanism, and signaling pathways (Supplementary file 1). Functional annotation by GO term enrichment analysis revealed that different sardine miRNA target proteins with molecular functions such as binding, transduction, and regulatory activity are involved in important biological processes such as cellular, developmental, metabolic, and reproductive processes (Fig. 2). Several experimental and computational studies have demonstrated that transcription factors are the major target molecules for various miRNAs (Barozai 2012) while for the proper functioning of the cells many miRNAs were reported to target signaling molecules (Hagen and Lai 2008). In this study important transcription factors targeted by sardine miRNAs include homeobox protein (*spi-miR-10a-5p*) that regulate gene expression and cell differentiation during early embryonic development and are involved in the regulation of patterns of anatomical development (morphogenesis) in both animals and plants (Bürglin and Affolter 2016); Wnt (*spi-miR-22a-3p*)-signaling cascade plays critical roles in embryonic patterning, cell fate determination, and tissue homeostasis (Van Noort and Clevers 2002); zinc finger proteins (*spi-miR-30b, spi-miR-152-3p, and spi-miR-734-3p*) are one of the most abundant groups of proteins and have a wide range of molecular functions including transcriptional regulation, ubiquitin-mediated protein degradation, actin targeting, DNA repair, cell migration, and numerous other processes (Cassandri et al. 2017); SRY-box 7 or SOX-7 (*spi-miR-143*) are transcription factors having critical roles in the regulation of diverse developmental processes in the animal kingdom and detected during embryonic development in many tissues, suggesting a role in differentiation and development (Takash 2001); and FEV (*spi-miR-1788-3p*), a member of one of the largest transcription factor family ETS. Among the important target signalling molecules Alpha kinase 2 (*spi-miR-122*), a member of Alpha kinase family are implicated in a large variety of cellular processes such as Mg2+ homeostasis, intracellular transport, cell migration, adhesion, and proliferation (Middelbeek et al. 2010); WD repeat-containing proteins (*spi-miR-489*) have critical roles in many biological functions such as signal transduction, transcription regulation and apoptosis (Li and Roberts. 2001); G protein-coupled receptor proteins (*spi-miR-34a*) are the largest family of membrane proteins and mediate most cellular responses to hormones and neurotransmitters, as well as being responsible for vision, olfaction and taste (Rosenbaum et al. 2009); Exportin 7 (*spi-miR-146b*) escorts multiple cytosolic proteins from the nucleus back into the cytoplasm, and thus may function to exclude numerous proteins that otherwise would interfere with gene expression if allowed to gather in the nucleus (Aksu et al. 2018); glutamate is the most abundant excitatory neurotransmitter in the vertebrate nervous system and one of the major functions of glutamate receptors (*spi-miR-7552-5p*) was found to be the modulation of synaptic plasticity, a property of the brain thought to be vital for memory and learning (Debanne et al. 2003); and Adenosine receptors (*spi-let-7a* and *spi-miR-143*) are reported to be involved in several key physiological processes, ranging from neuromodulation to immune regulation, and from vascular function to metabolic control (Chen et al. 2013) (Supplementary File 1).

## Conclusion

It is well established that several conserved, as well as species-specific miRNAs, are very crucial for different biological and metabolic pathways in animals as well as they can be used as biomarkers to monitor the effect of different anthropogenic stressors to the aquatic communities, especially for the teleost fishes, and hence it is important to profile miRNAs in non-model commercially as well as ecologically important teleost fishes to get an indication whether their conservation is required or not. Moreover, a number of brain-specific miRNAs from different marine animals already provided baseline information for commercial and conservation tasks. In this study, for the first time, using homology-based computational analysis and strict filtering criteria, 101 conserved miRNAs and 83 corresponding targets were identified in sardine fish. Among the predicted miRNAs, eight randomly selected miRNAs (*spi-miR9, spi-miR26, spi-miR128, spi-miR129, spi-miR132, spi-miR212, spi-miR219, and spi-miR338*) were validated and their quantitative expression revealed that all of them are brain enriched miRNAs corroborating some previous reports. Among the predicted miRNA targets, numerous targets were found to be involved in transcription and signaling pathways. Nonetheless, identification of miRNAs and their targets is the crucial step to initiate a miRNA-related study in a non-model animal species. Additionally, in the near future, current miRNA documentation may help in the creation of direct antimiRs and de-repressing specific targets in vivo to neutralize abiotic and biotic stress disorders in sardines. Nevertheless, we believe that our current study will be useful for strengthening the research on miRNA-mediated metabolic control in sardine and other fishes.

## Electronic supplementary material

Below is the link to the electronic supplementary material.

Supplementary file1 (DOCX 304 kb)

## Data Availability

The datasets during and/or analyzed during the current study are available from the corresponding author on reasonable request.
