# Supplementary material for: Characterization of miRNAs from sardine (Sardina pilchardus Walbaum, 1792) and their tissue-specific expression analysis in brain and liver
Source: 3 Biotech. 2020 Jun 26;10(7):318. doi: 10.1007/s13205-020-02298-y (PMC7320087; doi:10.1007/s13205-020-02298-y)
Supplement: Supplementary file 1 — Supplementary file1 (DOCX 304 kb) [file 13205_2020_2298_MOESM1_ESM.docx]

**
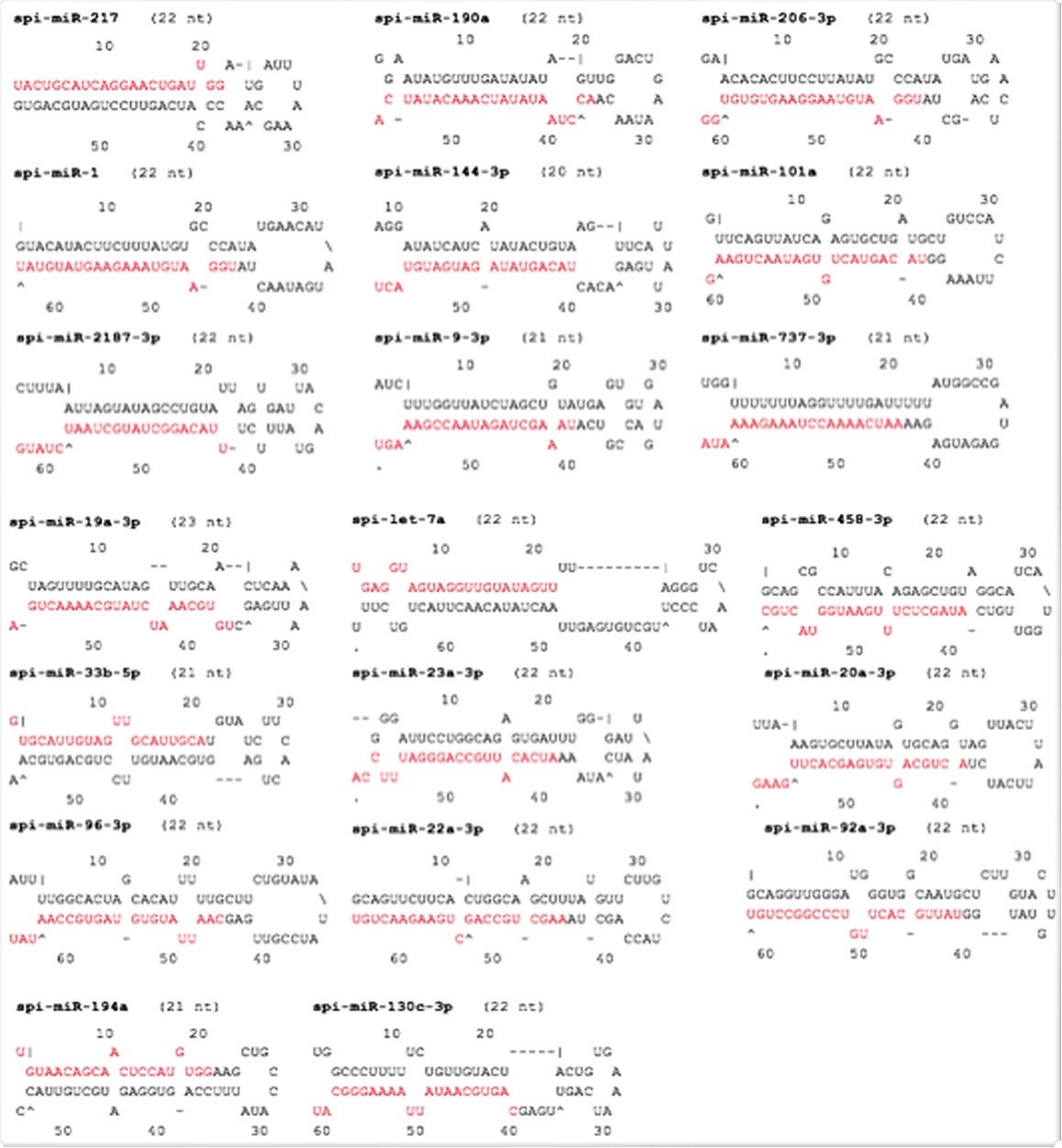
**

**Figure.** Stem-loop structures (top 20 structures with higher MFEI values are shown) of *Sardina pilchardus* microRNA precursors. Mature miRNAs are highlighted with red font.

**Table.** Potential targets of identified sardine microRNAs.

| miR Family | Name of Target Transcript | Molecular function | Biological process |
| --- | --- | --- | --- |
| let7 | Adenosine receptor A1-like | Receptor, transducer, G-protein couple receptor | Immune regulation, metabolic control |
| miR10 | Homeobox B4 | Transcription factor, DNA binding, developmental protein | Transcription, transcription regulation |
| miR15 | RNA-binding protein 20-like | RNA binding, zinc ion binding | Regulation of RNA splicing |
| miR16 | Potassium channel regulator | Ion channel | Ion channel, transport |
| miR19 | Oxysterol binding protein like 7 | Lipid binding, cholesterol binding, sterol binding | Lipid transport, transport |
| miR20 | Nucleotide binding oligomerization domain containing 1 | ATP-binding, nucleotide-binding | Immunity, innate immunity |
| miR22 | Wnt family member 10A | Transcription factor, Developmental protein | Wnt signaling pathway |
| miR24 | Dorsalis sec1 family domain containing 2 | - | Vesicle docking involved in exocytosis |
| miR25 | Protocadherin beta-16-like | Calcium ion binding | Hemophilic cell adhesion |
| miR26 | Tribbles homolog 2-like | ATP-binding, protein kinase activity | - |
| miR27 | Elongation factor for RNA polymerase II 2 | Ubiquitin protein ligase binding | Host-virus interaction, transcription, transcription regulation |
| miR30 | Zinc finger protein ZFAT-like | Transcription factor, Nucleic acid binding, zinc ion binding | - |
| miR31 | DnaJ heat shock protein family member C1 | Chaperone binding | Regulation of protein |
| miR33 | SH3 domain-binding protein 5-like | Guanine-nucleotide releasing factor | Intracellular signal transduction |
| miR34 | G protein-coupled receptor | G-protein coupled receptor, receptor, transducer, developmental protein | Apoptosis, cell cycle, differentiation, neurogenesis |
| miR92 | DBF4-type zinc finger-containing protein 2 homolog | Zinc ion binding, nucleic acid binding, | Cell cycle, DNA replication |
| miR93 | FAS associated factor family member 2 | Receptor | Apoptosis |
| miR100 | FUS RNA binding protein | DNA-binding, RNA binding, zinc ion binding, | Regulation of transcription, regulation of RNA splicing, RNA splicing |
| miR101 | Annexin A3-like | Calcium ion binding |  |
| miR103 | Glutaredoxin and cysteine rich domain containing 1 | Electron transfer activity | Receptor, cell redox homeostasis |
| miR122 | Alpha kinase 2 | Kinase, serine/threonine-protein kinase, transferase, ATP binding, nucleotide binding | Angiogenesis, regulation of autophagy |
| miR124 | Transmembrane protein 150A-like | - | - |
| miR125 | Sperm acrosome associated 6 | Lysozyme activity | Sperm egg recognition, immunity |
| miR126 | Calcitonin receptor | G-protein coupled receptor, receptor, transducer | Angiogenesis |
| miR128 | FSD1-like protein | - | - |
| miR129 | Matrix remodeling associated 5 | Extracellular matrix structural constituent | Response to transforming growth beta factor |
| miR130 | KIT proto-oncogene | Receptor tyrosine kinase, ATP binding, transferase, | Cell differentiation, cell adhesion, cell cycle |
| miR132 | Non-muscle caldesmon-like | Actin binding, myosin binding, calmodulin binding | Muscle contraction |
| miR133 | Glutamate ionotropic receptor delta type subunit 2 | Ion channel, receptor, | Ion transport, transport |
| miR135 | ER membrane protein complex subunit 9 | - | - |
| miR138 | Dehydrogenase/reductase SDR family member 13-like | - | - |
| miR140 | Olfactory receptor 11A1-like | G-protein coupled receptor, receptor, transducer | Olfaction, sensory transduction |
| miR142 | RIMS-binding protein 2-like | Receptor | Differentiation, spermatogenesis |
| miR143 | Adenosine receptor a2b-like  SRY-box 7 | G-protein coupled receptor, receptor, transducer  Transcription factor, DNA-binding | Angiogenesis, cell differentiation, |
| miR144 | Era like 12S mitochondrial rRNA chaperone 1 | RNA-binding, GTP binding, nucleotide binding | Ribosome biogenesis |
| miR146 | Exportin 7 | Nuclear export signal receptor activity | mRNA transport, protein transport, translocation, transport |
| miR148 | Fasciculation and elongation protein zeta 1 | Protein binding | Cell adhesion, transport, nervous system development |
| miR150 | BCL2 like 12 | Protein kinase and phosphatase binding, lipid binding | Apoptosis |
| miR152 | Zinc finger protein 90-like | Transcription factor, Nucleic acid binding, repressor, zinc ion binding | Transcription, transcription regulation |
| miR153 | Kelch repeat | - | Translation regulation |
| miR155 | Rab11a | Nucleotide binding, GTP binding | Cell cycle, protein transport, transport |
| miR183 | PiggyBAC transposable element-derived protein 4-like | Nucleic acid binding, zinc ion binding | - |
| miR184 | Vascular endothelial growth factor receptor 3 | Tyrosine protein kinase activity, ATP binding, nucleotide binding, developmental protein, receptor | Angiogenesis, new blood vessel formation, differentiation |
| miR187 | TLD domain-containing protein 2-like | - | - |
| miR190 | AN1-type zinc finger protein 5 | Transcription factor, DNA binding, zinc ion binding, | Skeletal system morphogenesis |
| miR192 | Ryanodine receptor 2 | Calcium channel, calmodulin binding, developmental protein, ion channel, receptor | Calcium transport, ion transport, transport |
| miR193 | Apolipoprotein B-100-like | Lipid binding, receptor | Lipid transport, lipoprotein metabolic process |
| miR194 | Germ cell-specific gene 1-like protein | - | Regulation of AMPA receptor activity |
| miR196 | Homeobox A9 | Developmental protein, DNA binding | Transcription, transcription regulation |
| miR199 | Integrin beta-3 | Integrin binding, protease binding, enzyme binding, receptor | Cell adhesion, cell migration |
| miR200 | Protein fem-1 homolog B-like | Death receptor binding | Apoptosis |
| miR203 | Kinesin-like protein KIF26A | ATP-binding, nucleotide binding, motor protein | Antigen processing, regulation of cell growth |
| miR204 | Hexokinase-2 | Allosteric enzyme, kinase, transferase, nucleotide binding, ATP-binding, glucose binding | Glycolysis |
| miR205 | Olfactory receptor class A-like protein 1  Vomeronasal type-1 receptor 4 | G-protein coupled receptor, receptor, transducer, pheromone binding  G-protein coupled receptor, receptor, transducer, pheromone receptor | Pheromone response, sensory transduction, olfaction  Pheromone response |
| miR206 | Ring finger protein 213 | DNA binding, repressor, transferase, zinc ion binding | Transcription, transcription regulator, transport, spermatogenesis |
| miR212 | Solute carrier family 6 member 2  GTPase imap family member 4-like | Protein kinase binding, potassium:chloride symporter  GTP-binding, nucleotide binding | Ion transport, transport, potassium transport, symport |
| miR214 | Dynamin-2-like  Zinc finger protein xlcof6-like | GTP binding, GTPase activity, nucleotide binding, motor protein, protein kinase binding  Transcription factor, Nucleic acid binding | Endocytosis, muscle fiber development, phagocytosis, spermatogenesis, cellular response |
| miR216 | 3 beta-hydroxysteroid dehydrogenase | Isomerase, multifunctional enzyme, oxidoreductase | Lipid metabolism, steroid metabolism, steroidogenesis |
| miR217 | Ankyrin repeat domain 44  Cytochrome p450 1a1-like | Developmental protein  Oxidoreductase, lyase iron, metal binding, heme binding | Differentiation, RNA-mediated gene silencing, spermatogenesis  Fatty acid metabolism, lipid biosynthesis, lipid metabolism, steroid biosynthesis |
| miR218 | Protein slit-like | Developmental protein, calcium ion binding, heparin binding | Differentiation, neurogenesis |
| miR219 | Diphosphoinositol pentakisphosphate kinase 1 | Kinase, transferase, ATP binding, nucleotide binding | Inositol phosphate metabolic process, inositol metabolic process |
| miR223 | Heme oxygenase 2 | Oxidoreductase, heme binding, iron binding, metal binding | Iron ion homeostasis |
| miR338 | Heme oxygenase 2 | Oxidoreductase, heme binding, iron binding, metal binding | Iron ion homeostasis |
| miR363 | Glypican-3-like | - | Cell migration, coronary vasculature development |
| miR429 | Von Willebrand factor A domain-containing protein 7-like | Developmental protein | Nervous system development |
| miR430 | Mucin-2-like | - | Maintenance of gastrointestinal epithelium, O-glycan processing |
| miR455 | Doublesex- and mab-3-related transcription factor A2-like | DNA binding, metal ion binding | Transcription, transcription regulation |
| miR456 | GTPase Era | GTP binding, RNA binding, nucleotide binding | Ribosome biogenesis, |
| miR458 | Extended synaptotagmin-1-like  Phospholipase c gamma 2 | Lipid binding, metal ion binding, calcium ion binding  Hydrolase, transducer, calcium ion binding | Lipid transport, transport  Lipid degradation and metabolism |
| miR459 | Caskin-1-like | Identical protein binding | Signal transduction |
| miR460 | Hemicentin-1-like | Calcium ion binding | Fin development, fin morphogenesis |
| miR489 | Peptidylprolyl isomerase domain and WD repeat containing 1 | Isomerase | mRNA processing, mRNA splicing |
| miR724 | Copine-5-like | Calcium dependent phospholipid binding | Cellular response to calcium ion |
| miR726 | Red-sensitive opsin-like | G-protein coupled receptor, photoreceptor protein, receptor, retinal protein, transducer | Visual perception, phototransduction, sensory transduction, vision |
| miR727 | CD44 antigen-like  Syntrophin beta 2 | Hyaluronic acid binding, collagen binding, receptor, blood group antigen  Actin binding, RNA binding, calmodulin binding | Cell adhesion |
| miR728 | Acidic leucine-rich nuclear phosphoprotein 32 family member E-like | Histone binding, chaperone, chromatin regulator | Histone exchange |
| miR734 | Zinc finger protein 292-like | Transcription factor, Nucleic acid binding, zinc ion binding | RNA splicing, transcription, transcription regulation |
| miR1788 | FEV transcription factor  Mastermind-like protein 3 | Transcription factor,  DNA binding, developmental protein  Activator | Cell differentiation, transcriptional regulator, neurogenesis  Transcriptional regulator, notch signaling pathway |
| miR2187 | Intraflagellar transport 172 | Developmental protein | Retina development |
| miR2188 | Mediator complex subunit 1 | Activator | Transcription regulation |
| miR7552 | Glutamate receptor ionotropic, delta-1-like | Ionotropic glutamate receptor activity, ion channel, receptor | Ion transport, transport |
| miR8160 | Round spermatid basic protein 1 | Chromatin regulator, oxidoreductase, metal ion binding | Chromatin organization |
| miR10545 | Stabilin 2 | Calcium ion binding, hyaluronic acid binding | Blood vessel development, cell adhesion |
